# MANF Is Neuroprotective in Early Stages of EAE, and Elevated in Spinal White Matter by Treatment With Dexamethasone

**DOI:** 10.3389/fncel.2021.640084

**Published:** 2021-07-07

**Authors:** Jinhan Nam, Tapani K. Koppinen, Merja H. Voutilainen

**Affiliations:** Division of Pharmacology and Pharmacotherapy, Faculty of Pharmacy, University of Helsinki, Helsinki, Finland

**Keywords:** MS, EAE, MANF, dexamethasone, neuroinflammation, UPR, Reactive glia

## Abstract

Multiple sclerosis (MS) is a progressive autoimmune disease characterized by T-cell mediated demyelination in central nervous system (CNS). Experimental autoimmune encephalomyelitis (EAE) is a widely used *in vivo* disease model of MS. Glucocorticoids such as dexamethasone (dex) function as immunosuppressants and are commonly used to treat acute exacerbations of MS. Dex is also often used as a positive control in EAE studies, as it has been shown to promote motor behavior, inhibit immune cell infiltration into the CNS and regulate the activation of glial cell in EAE. This study further validated the effects of intravenously administrated dex by time-dependent fashion in EAE. Dex postponed clinical signs and motor defects in early stages of EAE. Histological analysis revealed that the degeneration of myelin and axons, as well as the infiltration of peripheral immune cells into the white matter of spinal cord was inhibited by dex in early stages of EAE. Additionally, dex-treatment delayed the neuroinflammatory activation of microglia and astrocytes. Furthermore, this study analyzed the expression of the neurotrophic factor mesencephalic astrocyte-derived neurotrophic factor (MANF) in EAE, and the effect of treatment with dex on MANF-expression. We show that in dex-treated EAE mice expression MANF increased within myelinated areas of spinal cord white matter. We also show that intravenous administration with hMANF in EAE mice improved clinical signs and motor behavior in the early stage of EAE. Our report gives insight to the progression of EAE by providing a time-dependent analysis. Moreover, this study investigates the link between MANF and the EAE model, and shows that MANF is a potential drug candidate for MS.

## Introduction

Multiple sclerosis (MS) is a chronic demyelinating autoimmune disease of the central nervous system (CNS). It affects over 2.8 million patients worldwide (Reich et al., 2018). Its pathogenesis involves the activation of both peripheral and glial immune systems as a response to sensitization to endogenous myelin, resulting in infiltration to the CNS by peripheral mononuclear cells and an increase in pro-inflammatory signaling in both astrocytes and microglia in the CNS (Correale and Farez, 2015; Chu et al., 2018; Ponath et al., 2018). Experimental autoimmune encephalomyelitis (EAE) is one of the oldest neurological disease models in existence and is widely used to screen pharmacological compounds searching for drug candidates for MS. It involves the immunization of a target animal’s immune system against myelin via the injection of myelin components, such as myelin basic protein (MBP), proteolipid protein (PLP) or myelin oligodendrocyte glycoprotein (MOG) (Ransohoff, 2012). This immunization results in a CD4^+^ T-cell driven inflammatory attack on the CNS, which induces paralytic demyelination. EAE is a very flexible model, and can be adjusted to utilize different animal species, immunizing components, target cell types and disease progressions. The most common model remains the C57BL/6 mouse EAE model, which features immunization with residues 35–55 of the MOG peptide emulsified in Freund’s adjuvant, combined with injections with pertussis toxin. This results in a reproducible, monophasic disease course (Ransohoff, 2012). EAE models have in a few cases been used successfully in the preclinical development of MS therapeutics (Yednock et al., 1992; Brinkmann et al., 2002), but have generally been a poor predictor of drug efficiency (Steinman and Zamvil, 2006; Constantinescu et al., 2011; Lassmann and Bradl, 2017).

Shortcomings in predictiveness are believed to be caused by key differences in the pathology of MS and EAE. While pathology in MOG35–55/PTX immunized EAE mice is driven by CD4^+^ T cells, and B cells play a very minor role, clonally expanding CD8^+^ T cells dominate lesions in MS and targeting B cells has been therapeutically successful (Babbe et al., 2000; Hauser et al., 2008; Kappos et al., 2011). On the other hand, MS is not induced by a single antigen, and immunization with different components can produce varied immune responses. Immunization with the MP4 MOG-PLP fusion protein, for example, shows more involvement by B cells and CD8^+^ T cells than single-antigen immunized EAE models (Kuerten et al., 2006).

MS cannot be cured. It is currently only treated with immunomodulatory drugs that either reduce the number of peripheral immune cells infiltrating the CNS, or shift their phenotype toward a more anti-inflammatory state (Faissner and Gold, 2018). Corticosteroids (CSs) such as methylprednisolone and dexamethasone (dex) are used as first-line therapies during acute exacerbations (Smets et al., 2017). The anti-inflammatory mechanism of CSs is mediated through a binding to glucocorticoid nuclear hormone receptors located in the cytosol, leading to the translocation of the activated receptor to the nucleus, where it regulates glucocorticoid response elements (GREs). The activated receptors also directly inhibit general pro-inflammatory cytokine pathways, such as nuclear factor-kappa B (NF-κB). CSs can additionally bind to cell membrane components, inducing rapid anti-inflammatory non-genomic transduction cascades (Panettieri et al., 2019). Dex is often utilized as a positive control in EAE studies, because it reliably reduces motor function loss, delays the peak of the disease, preserves myelin, and inhibits immune cell infiltration (Donia et al., 2010; Dos Santos et al., 2019).

Neurotrophic factors (NTFs) play an important role in CNS, regulating cellular processes such as differentiation and proliferation. In states of disease, they show protective and restorative effects. In EAE mice, knocking out CNTF (ciliary neurotrophic factor) increases oligodendrocyte apoptosis and negatively influenced disease progress and recovery compared to the process in wild type mice (Linker et al., 2002). Several NTFs have been considered as therapeutic agents for neurological disease and injury (Pöyhönen et al., 2019), including mesencephalic astrocyte-derived neurotrophic factor (MANF) (Petrova et al., 2003), which has been reported to possess protective and restorative effect in the peripheral nervous system (PNS) and CNS in disease animal models (Voutilainen et al., 2009; Lindahl et al., 2014; Mätlik et al., 2018). The association and potential therapeutic of MANF in MS and its disease models has not been investigated before.

Our report characterizes the effect of intravenously administered dex on the pathological mechanisms and progression of EAE by providing a time-dependent analysis of its impact on neuroinflammation and demyelination in the C57BL/6 model. It uncovers a regulation of MANF by dex during EAE and investigates the time-dependent expression of MANF in EAE mice. It is also the first examination of the effects of treatment with MANF on disease progression and motor behaviors in the MOG_3__5__–__5__5_/CFA immunized EAE mice model.

## Materials and Methods

### Animals

Mice utilized in this experiment were C57BL/6 obtained from Envigo. Upon initiation of the experiment, the male mice were 7 weeks old and weighed approximately 25 g. Animal were housed under a 12:12 light:dark cycle. Water and food were available *ad libitum*. Experiment groups divided in the following manner: for effects of dex in a time-dependent manner, naïve control (*n* = 7), EAE + vehicle (*n* = 23), and EAE + Dex (*n* = 26); for study into treatment with human MANF, EAE + vehicle (*n* = 10), EAE + hMANF 1.5 μg (*n* = 10), and EAE + hMANF 3 μg (*n* = 9); for study into effects of dex in naïve mice, naïve controls (*n* = 6), Dex (*n* = 21).

### EAE Induction

EAE was induced using the EAE induction kit for C57BL/6 mice (EK-2110, Hooke Laboratories). Induction of EAE was performed according to kit instructions. Mice were subcutaneously injected into the upper and lower backs with MOG_3__5__–__5__5_ peptides emulsified with complete Freund’s adjuvant (CFA) emulsion. Additionally, mice received 200 ng of pertussis toxin (PTX, kit component) intraperitoneally (i.p.) at 2 and 24 h after MOG_3__5__–__5__5_/CFA injection. For naïve mice, CFA-MOG/PTX was replaced with PBS. The clinical scores and body weight of mice were evaluated daily. Clinical scoring followed a scale of (0)–(5), in which (0) represents no apparent motor symptoms or paralysis, (1) represents a completely limp tail, (2) represents weakened hind legs, (3) represents completely paralyzed hind legs, (4) represents partial front leg paralysis, and (5) represents death from paralysis. Mice that were scored at 2.0 or higher were provided with cotton bedding and soft food. Mice that were scored over 3.0 received 0.5 ml of saline to recover from dehydration. Treatments were given to mice as 100 μl injections in the tail vein (i.v.) every second day, starting on day 2 after EAE induction, under isoflurane anesthesia (4% induction, 2–3% maintenance). Dex groups received a dose of approximately 4.3 mg/kg of dex (D1159, Sigma) from 2 to 16 dpi, and 5 mg/kg from 16 to 28 dpi. This dose was calculated based on mean group weights, and Supplementary Figure 1 represents the raw data for body weight of each group. Other groups were given either human MANF (1.5 or 3 μg, Cat: P–101–100, icosagen, Estonia) dissolved in PBS (B. Braun Medical, Finland), or vehicle (PBS).

### Analysis of Motor Behavior

The rotarod (Ugo Basile) and open field (ENV-520, Med Associates Inc.) tests were carried out to investigate motor function deficit. In the rotarod test, mice were trained once a day for 3 days on an accelerating rotating platform (from 4 to 40 rpm for a maximum of 300 s). Once mice fell off from the rotating rod, the latency to fall (seconds it takes for the mouse to drop from the rod) was recorded. After training, experimental groups were divided based on latency to fall. After EAE induction, mice were tested at an interval of 7 days. In the open field-test, mice were trained once in the locomotor activity chamber for 1 h before EAE induction. After EAE induction, travel distance and rearing activity of mice in the open-field chamber was measured for 1 h at an interval of 7 days.

### Immunohistochemistry

Mice were anesthetized by sodium pentobarbital (Mebunat, Orion Pharma, Finland) and transcardially perfused with phosphate-buffered saline (PBS) on days 7, 14, 21, and 28 after induction. Following perfusion with PBS, lumbar spinal cords were extracted and post-fixed in 4% paraformaldehyde (PFA, Sigma) for 48 h. Samples were then paraffinized and embedded in paraffin and sectioned into 16 μm transverse (spinal cord) slices placed on glass slides. Before immunohistochemical staining, the slides were deparaffinized using xylene and ethanol. Antigen retrieval was performed by heating slides in a sodium citrate buffer (10 mM Sodium citrate, pH 6.0, 0.05% Tween-20).

#### For DAB-Immunostaining

Tissues were processed with deparaffinization and antigen retrieval. Endogenous peroxidases were inactivated by Peroxidase blocking solution (200 ml TBS mixed with 3.75 ml of 30% H_2_O_2_) for 30 min at room temperature. After washing three times with TBS to remove peroxidase blocking solution, samples were incubated with blocking solution (TBS with 0.1% Tween-20 and blocked with 1.5% normal goat or horse serum) for 1 h at room temperature. After blocking, slides were incubated overnight at 4°C with primary antibodies (Rabbit anti-Iba1 1:1,000,01-19741, Wako, Japan; Goat anti-GFAP 1:400, ab53554, Abcam; Mouse anti-MBP 1:520, sc-271524, Santa Cruz, United States; Rat anti-mouse CD45 1:500, 103101, Biolegend, United States), diluted in the blocking solution. The following day, tissues were washed with TBS-T three times, then incubated with corresponding biotinylated secondary antibodies (Vector Laboratories, United States) diluted in blocking solution for 1 h at room temperature. Tissues were washed with TBS-T, incubated with an avidin-biotin complex solution for 1 h, and the resulting signal was visualized using 3, 3′-diaminobenzidine (DAB, Vector Laboratories Inc., United States). Slides were scanned using a Pannoramic 250 (3DHistech, Hungary) slide scanner with 20× magnification and single layer focus. Scanned slides were processed and captured images using Pannoramic Viewer 1.15.4 (3DHistech).

#### Immunofluorescent Staining

Tissues were permeabilized with TBS-T (TBS with 0.1% Tween-20) and blocked with 5% bovine serum albumin (BSA, Sigma) for 1 h. After blocking, tissues were incubated overnight 4°C with primary antibodies (Mouse anti-MBP 1:520, sc-271524 Santa Cruz; Rabbit anti-Neurofilament 1:750, ab1987 Millipore, United States; Rabbit anti-MANF 1:1,000, LS-B2688, LSBio, United States; Goat anti-TPPP, 1:500, PA5-19243, Invitrogen, United States) diluted in blocking solution. The next day, tissues were washed three times with TBS-T, and incubated for 1 h with fluorescence-conjugated corresponding secondary antibodies (Alexa 488, 568, and 647, 1:200, donkey anti-rabbit, anti-goat, and anti-mouse, Thermo Fisher Scientific), diluted in blocking solution at room temperature. Tissues were washed and incubated with DAPI to visualize nuclear and counterstain (1:1,000, Thermo Fisher Scientific). Laser scanning confocal micrographs of the fluorescently labeled lumbar spinal cords were acquired with an LSM700 (Carl Zeiss) using a LCI Plan-Neofluar 63×/1.3 Imm Corr objective with 80% glycerol.

### Inflammation Scores

The inflammation scores were evaluated using hematoxylin-eosin stained (H&E) images. Tissues were stained with H&E to visualize infiltrated cells in spinal cords and were scanned with a Pannoramic 250 (3DHistech, Budapest, Hungary) slide scanner with 20× magnification and single layer focus. The infiltration of peripheral immune cells was scored according to an inflammatory scoring setup described earlier: (0) no inflammation; (1) cellular infiltrate only detected in the perivascular areas and meninges; (2) mild cellular infiltrate in white matter areas of spinal cords; (3) moderate cellular infiltrate in white matter areas of spinal cords (4) severe cellular infiltrate in the whole white matter of spinal cords (Okuda et al., 2002).

### Image Analysis

Image analysis was performed using Fiji image J. With tissues DAB-stained against Iba1 or GFAP images were captured at 20**×** magnification and stained areas (pixel value) were quantified from four to six images per animal. For CD45, CD45-positive cells were from four images per animal. With tissues stained using immunofluorescent antibodies, four fluorescence images were captured in the white matter of the spinal cords at 63**×** magnification. The pixel area of each target protein was quantified and compared against the mean result from the naïve control group. The colocalization of MANF and MBP or MANF and TPPP was evaluated using the colocalization plug-in (Nam et al., 2015).

### Statistical Analysis

All values were presented as mean ± standard error of the mean (SEM). Statistical significance was determined by unpaired *t*-tests for single comparisons, or two-way ANOVA followed by Tukey’s *post hoc* test or Sidak’s *post hoc* test for multiple comparisons. All statistical analysis was performed using GraphPad8 Prism software (GraphPad Software, United States).

## Results

### Dexamethasone Postpones Clinical Signs and Ameliorated Motor Behavior Dysfunction in EAE

**FIGURE 1:**
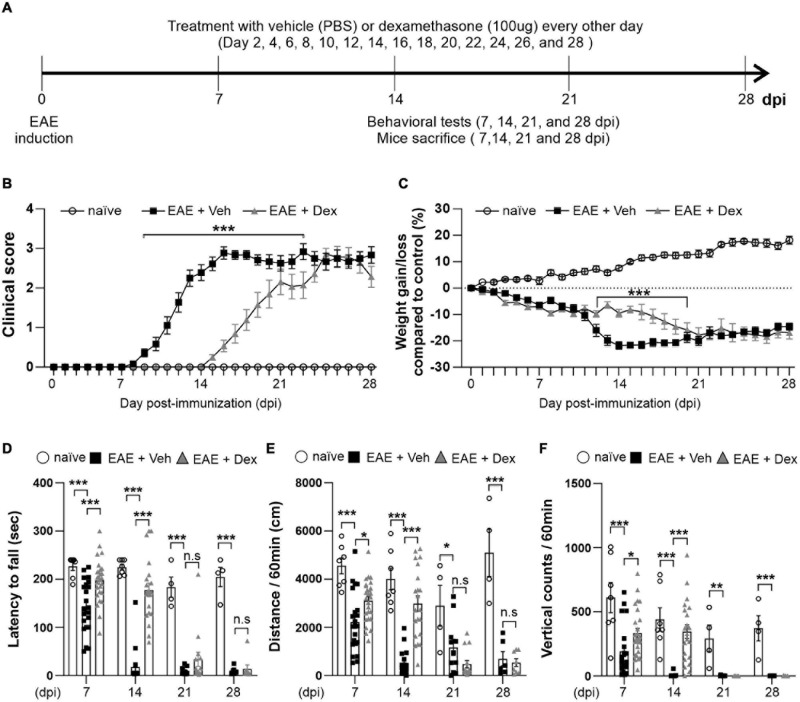
Dexamethasone delayed the manifestation of clinical signs and reduced motor behavior dysfunction in EAE mice. **(A)** Diagram of the experiment design. To induce EAE, C57BL/6 were immunized with MOG_3__5__–__5__5_/CFA and Pertussis toxin (PTX). EAE mice received dexamethasone (100 μg) or vehicle (PBS) intravenously (i.v. tail vein) every second day. Mice were transcardially perfused after behavioral experiments at an interval of 7 days. **(B)** Mean clinical scores for each day from immunization (0 dpi) to 28 dpi. At ***P < 0.001, treatment with dexamethasone significantly delayed the manifestation of clinical signs compared to the EAE + Veh group at 9–22 dpi. Mean ± SEM, *n* = 4–26. **(C)** Gain/loss of body weight. At ***P < 0.001, treatment with dexamethasone significantly reduced body weight loss compared to the EAE + Veh group at 12 dpi to 20 dpi. Mean ± SEM, *n* = 4–26. **(D)** Latency to fall during the Rotarod test was recorded at 7, 14, 21, and 28 dpi. **(E)** Distance traveled and **(F)** vertical counts (rearing-up) was recorded with the open-field test at 7, 14, 21, and 28 dpi. **(D,E)** n.s; non-significant, *P < 0.05, **P < 0.01, and ***P < 0.001, Mean ± SEM. *n* = 7–27 (7 dpi), *n* = 7–21 (14 dpi), *n* = 4–14 (21 dpi), and *n* = 4–8 (28 dpi). **(B–F)** 2-way ANOVA was used followed by Tukey’s *post hoc* test for multiple comparisons. Data from 4 repeated experiments.

To explore the effect of dex in EAE, C57BL/6 immunized with MOG_3__5__–__5__5_ underwent a 28-day testing period as described by the experimental design timeline in Figure 1A. Before the induction of EAE, mice underwent testing with the rotarod behavioral test for 3 days, based on which mice were balanced into treatment groups (naïve, EAE + Veh and EAE + Dex). We observed first clinical signs (clinical score 0.5–1.0) in EAE + Veh mice at 8 days post-immunization (dpi). The disease course reached its peak (clinical score 3.0–4.0) at 14 dpi, and continued until 28 dpi, at which point the last animals were sacrificed. Animals in the naïve group did not display clinical signs. Animals in the EAE + Dex group showed onset of clinical signs at 14 dpi and reached peak clinical scores at 23 dpi. Thus, dex-treatment in EAE mice postponed the onset of EAE by an average of 6 days compared to EAE + Veh mice (Figure 1B). Gain or loss of body weight is another clinical sign of the EAE mice model: the body weight for both EAE induced groups was significantly reduced compared to the naïve group from 4 dpi onward, while the naïve group increased in body weight throughout the experiment. The loss of body weight before the onset of the disease may be partially caused by chronic stress caused by repeated iv injections, as it occurs in both EAE groups despite different delayed onset in dex-treated animals. From 11 to 18 dpi, dex-treated mice lost significantly less weight than the EAE + Veh group (Figure 1C).

Rotarod and open-field tests are widely used to evaluate motor coordination in rodent models of CNS disease, including EAE (Dutra et al., 2013). To study motor behavior, we carried out the Rotarod and open-field testing at 7, 14, 21, and 28 dpi (Figure 1A). The Rotarod test for EAE + Veh mice showed robust motor deficits at 7 dpi, and near total performance loss at 14, 21 and 28 dpi. In contrast, treatment with dex inhibited loss of motor function at 7 and 14 dpi, but not at 21 and 28 dpi (Figure 1D). In parallel with rotarod behavior, the total travel distance and the number of vertical counts (representing mice rearing up) were significantly decreased in EAE + Veh mice at 7, 14, 21, and 28 dpi. Treatment with dexamethasone improved travel distance and vertical counts significantly at 7, 14 dpi (Figures 1E,F), but had no effect at 21 and 28 dpi.

### Dexamethasone Delays Myelin and Axon Loss, as Well as Peripheral Immune Cell Infiltration at Early Stages of EAE

**FIGURE 2:**
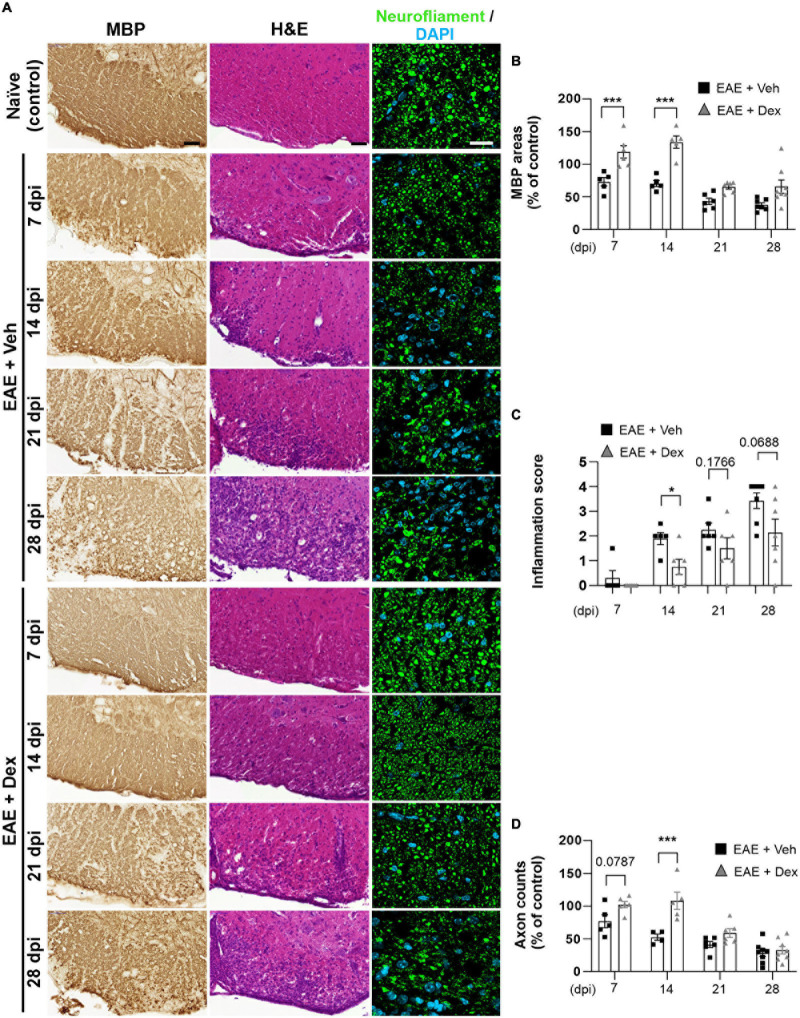
Dexamethasone inhibited myelin and axon loss, as well as immune cell infiltration in the early stage of EAE. **(A)** Representative images from MBP (Left), Hematoxylin-Eosin (H&E, Center), and neurofilament (Right; Green: Neurofilaments, Cyan: DAPI nuclear stain) immunostained mouse spinal cords at 7, 14, 21, and 28 dpi. **(B)** Quantification of MBP area at each time point. ***P < 0.001, Mean ± SEM. *n* = 5–8 per each group. **(C)** Evaluation of inflammation score from hematoxylin-eosin staining. *P < 0.05, **P < 0.01, and ***P < 0.001, Mean ± SEM, significance base on unpaired *t*-test. *n* = 5–7 per each group. **(D)** Quantification of the number of neurofilament-positive axons. ***P < 0.001, Mean ± SEM. *n* = 5–7 per each group. **(B,D)** 2-way ANOVA followed by Sidak’s *post hoc* test for multiple comparisons. Scale bar; 50 μm for MBP, H&E. 20 μm for neurofilament. Data from 4 repeated experiments.

Infiltration of immune cells to the CNS across the blood-brain-barrier is the primary driver of demyelination and neurodegeneration in EAE (Reich et al., 2018). To observe the effects of dex-treatment on the immunopathogenesis of EAE in a time-dependent manner, animals from each group were sacrificed at 7, 14, 21, and 28 dpi, and their lumbar spinal cords visualized using various immunohistochemical methods (Figure 2A). MBP-positive surface areas were shown to significantly decrease in a time-dependent manner in EAE + Veh animals compared to naïve controls. In contrast, dex-treatment in EAE mice protected lumbar spinal cords against demyelination at 7 and 14 dpi, but not at 21 and 28 dpi (Figures 2B, 4A).

**FIGURE 3:**
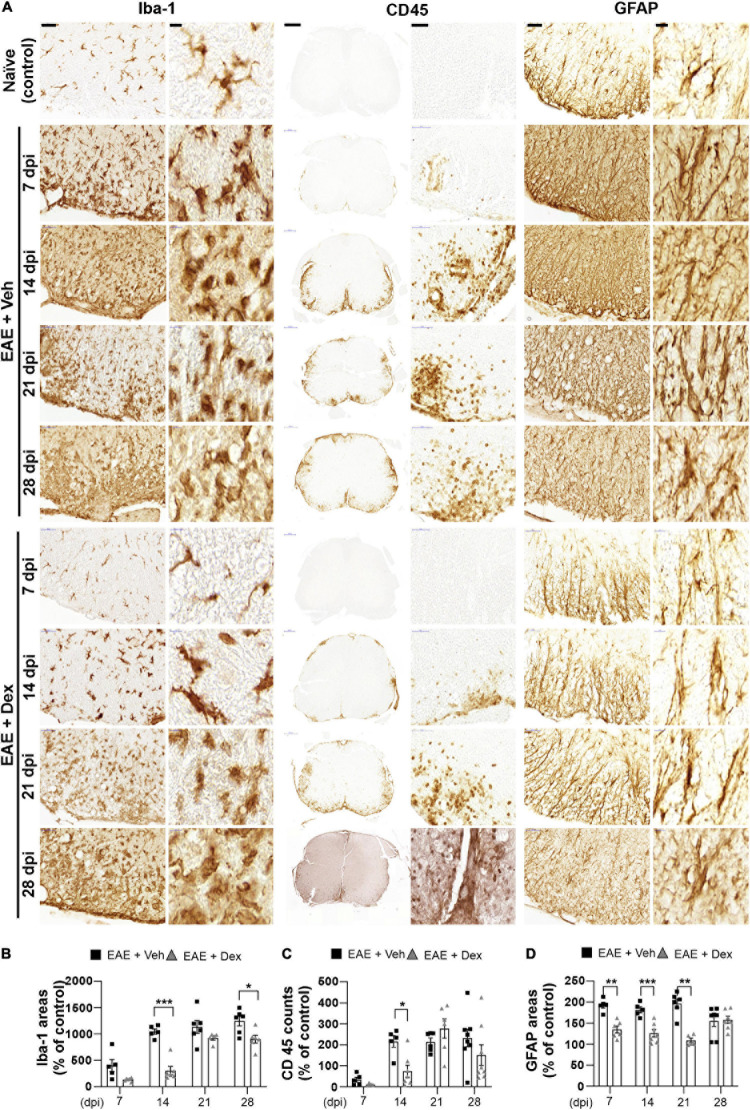
Dexamethasone attenuated neuroinflammatory activation of microglia, leukocytes, and astrocytes during the early stage of EAE. **(A)** Representative images for Iba-1 (Left), CD45 (Center), and GFAP (Right) from immunostained mouse spinal cords at 7, 14, 21, and 28 dpi. **(B)** Quantification of Iba-1 area in each time point. *P < 0.05, and ***P < 0.001, Mean ± SEM. *n* = 5–6 per each group. **(C)** Quantification of the number of CD45-positive cells in each time point. *P < 0.05, and **P < 0.01, Mean ± SEM. *n* = 5–8 per each group. **(D)** Quantification of GFAP area in each time point. **P < 0.01, and ***P < 0.001, Mean ± SEM, *n* = 5–7 per each group. **(B–D)** 2-way ANOVA followed by Sidak‘s *post hoc* test for multiple comparisons. Scale bars: 200 μm for CD45; 50 μm for Iba-1, GFAP; 10 μm for enlarged Iba-1, GFAP and CD45. Data from 4 repeated experiments.

**FIGURE 4:**
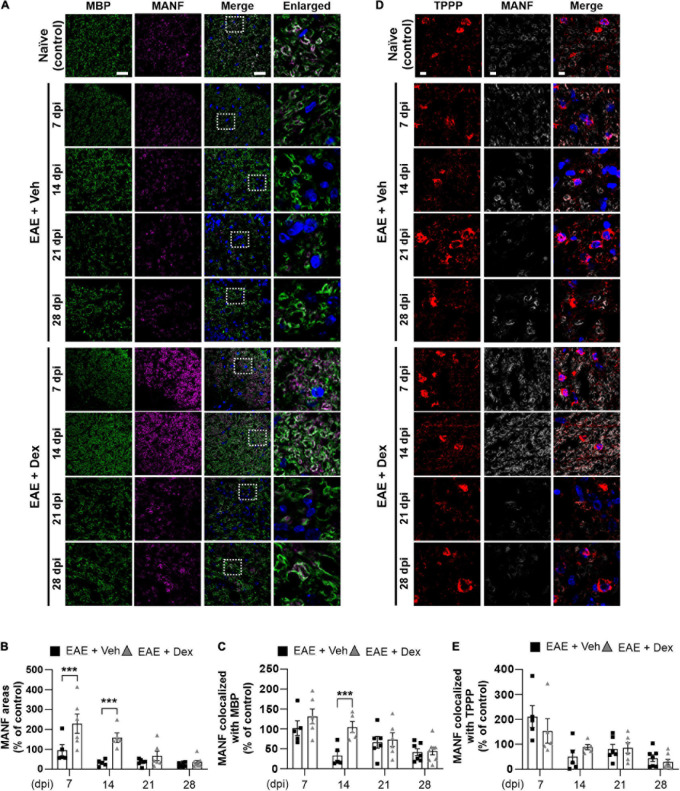
MANF levels within myelin sheaths, but not the cell bodies of mature oligodendrocytes, were increased by dexamethasone in the early stage of EAE. **(A)** Representative fluorescence images of MBP (Green), MANF (Magenta), and DAPI (Blue) in the white matter of mouse spinal cords at 7, 14, 21, and 28 dpi. Enlarged images were taken from boxed areas within merged images. **(B)** Quantification of MANF area in each time point. ***P < 0.001, Mean ± SEM. *n* = 5–8 per each group. **(C)** Quantification of MANF-expression area within MBP-positive areas at each time point. ***P < 0.001, Mean ± SEM, *n* = 5–7 per each group. **(D)** Representative fluorescence images of TPPP (Red), MANF (Light gray), and DAPI (Blue) in the white matter of mouse spinal cords at 7, 14, 21, and 28 dpi. **(E)** Quantification of MANF-expression area within TPPP-positive areas at each time point. *n* = 5–8 per each group. **(B,C,E)** 2-way ANOVA followed by Sidak’s *post hoc* test for multiple comparisons. Scale bars: 200 μm for MBP, MANF, and Merge; 10 μm for enlarged imaged. Data from 4 repeated experiments.

A small degree of infiltration by peripheral immune cells in EAE + Veh mice was detected at 7 dpi, with inflammatory scores increasing each week throughout the experiment. In EAE mice treated with dex, infiltration was first detected at 14 dpi and increased progressively through the end of the experiment, showing a trend consistent with the concept of dex delaying disease onset by a week (Figure 2C). Similarly, EAE + Veh animals showed a progressive loss of neurofilament-positive axons, whereas treatment with dex preserved axons at 7 and 14 dpi, before the onset of rapid axon loss at 21 and 28 dpi (Figure 2D).

### Dexamethasone Delays the Neuroinflammatory Activation of Microglia, Leukocytes, and Astrocytes

The neuroinflammatory activation of resident glial cells is thought to contribute to neurodegeneration in EAE (Matsumoto et al., 1992). Visualization of Iba1 in lumbar spinal cords by DAB-stain showed a progressive increase in neuroinflammatory microglial activation throughout the disease, unlike in naïve controls. In mice treated with dex this increase was delayed at 7 and 14 dpi, but microglial activation reached similarly high levels at later timepoints (Figures 3A,B). The distribution of leukocytes was determined by analyzing slides stained for leukocyte common antigen CD45. As with Iba-1 immunostaining, increased counts of cell positive for CD45 were detected at 7 dpi (though still small), reached peak levels at 14 dpi, and remained at peak levels until the end of experiment, compared to naïve controls. The cell counts of CD45-positive cells in mice treated with dex were small at 7 dpi, and significantly smaller than in EAE + Veh mice at 14 dpi (Figures 3A,C). By staining for GFAP, an intermediate filament-III protein that is upregulated in astrocytes during neuroinflammation, a sustained increase in astrocyte activation could be detected in EAE + Veh animals throughout the disease. This increase was significantly ameliorated by dex-treatment at 7, 14, and 21 dpi (Figures 3A,D).

### Dexamethasone Significantly Boosts MBP-Positive Myelin Sheath MANF-Levels During Early Stages of EAE

NTFs play an important role in neuroinflammation. Therapeutic potentials of NTFs such as CNTF have been studied using the EAE mouse model (Linker et al., 2002). MANF, an endogenous protein with NTF-properties known to possess neuroprotective effects in animal models of Parkinson’s disease (PD) and diabetes (Voutilainen et al., 2009; Lindahl et al., 2014; Pöyhönen et al., 2019), has not been studied using EAE before. In order to investigate whether MANF is associated with EAE, we performed a double staining in lumbar spinal cords against MBP and MANF. We found that dex-treatment during EAE increased MANF-positive areas significantly in the white matter compared to the EAE + Veh group at 7 and 14 dpi, but this effect was progressively reduced at later time points (Figures 4A,B). MANF-positive areas in EAE animals remained at similar levels as those in naïve controls at 7 dpi, but were dampened at 14, 21, and 28 dpi. Similarly, analysis of colocalization showed that MANF colocalized with MBP in both EAE + Veh and EAE + Dex groups at the same levels as naïve controls at 7 dpi, but this colocalization was reduced at all later timepoints in the EAE + Veh group (Figures 4A,C). The colocalization of MANF and MBP in dex-treated mice was significantly higher than in EAE + Veh mice at 14 dpi and at similar levels as the EAE + Veh group at 21 and 28 dpi. The colocalization of MANF with the mature oligodendrocyte cell body marker TPPP was not affected by treatment with dex indicating that dex only increased MANF-levels in myelinated axons, the primary targets of autoimmunity in EAE (Figures 4D,E).

In order to test whether dex affects the expression of MANF in naïve mice, we treated healthy C57BL/6 mice with 100 μg of dex i.v., and sacrificed them 0.5, 1, 3, and 6 h later (Supplementary Figure 2). Treatment with dex did not show a statistically significant increase in either MANF or MBP surface area, suggesting that the boosting effect of dex on MANF occurs specifically during the early stages of EAE.

Endoplasmic reticulum (ER) stress and the unfolded protein response (UPR) play important role in EAE, contributing to oligodendrocyte death and glial activation (Lin and Popko, 2009; Reverendo et al., 2019). MANF, a UPR-induced protein with NTF-properties, is believed to be expressed in tissues such as the spinal cord white matter as a response to intense ER stress (Danilova et al., 2019). If MANF is an indicator of ER stress, it would be reasonable to assume that dex increased expression of MANF by inducing ER stress in the earlier timepoints of the disease. However, analysis of the expression of GRP78, an upstream regulator of UPR, in lumbar white matter showed no difference between EAE and dex groups in UPR activation (Supplementary Figure 3).

### MANF Delays Disease Progression and Protects Motor Behavior in Early Stages of EAE

**FIGURE 5:**
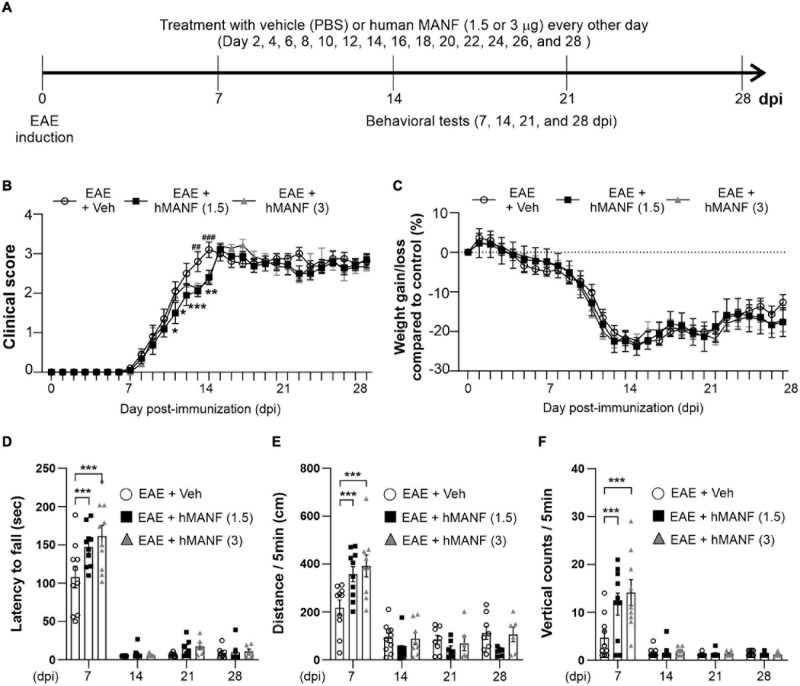
MANF ameliorated clinical signs and reduced motor behavior dysfunction during the early stage of EAE. **(A)** Diagram of the experiment design. To induce EAE, C57BL/6 were immunized with MOG_3__5__–__5__5_/CFA and Pertussis toxin (PTX). Mice received human MANF (1.5 or 3 μg) or PBS as a vehicle control intravenously (i.v. tail vein) every second day. Mice performed behavioral tests at an interval of 7 days. **(B)** Mean clinical scores for each day from immunization (0 dpi) to 28 dpi. At *P < 0.05, **P < 0.01, and ***P < 0.001, treatment with hMANF (1.5 μg) significantly delayed clinical signs manifestation compared to the EAE group at 11 dpi to 14 dpi, ^##^*P* < 0.01, and ^###^*P* < 0.001, treatment with hMANF (3 μg) significantly delayed clinical signs manifestation compared to the EAE group at 13 and 14 dpi. Mean ± SEM, *n* = 9–10. **(C)** Gain/loss of body weight. Mean ± SEM, *n* = 9–10. **(D)** Latency to fall during the Rotarod test was recorded at 7, 14, 21, and 28 dpi. **(E)** Distance traveled and vertical counts (rearing-up) was recorded with the open-field test at 7, 14, 21, and 28 dpi. **(D–E)** ***P < 0.001, Mean ± SEM. *n* = 9–10. **(B–F)** 2-way ANOVA was used followed by Tukey’s *post hoc* test for multiple comparisons. Data from a single independent experiment.

In order to study the role of MANF in EAE, another 28-day testing period was performed on C57BL/6 mice immunized with MOG_3__5__–__5__5_ (Figure 5A), replacing dex with human MANF. While all animals showed a similar disease onset at day 8, mice that were treated with either 1.5 or 3 μg of hMANF i.v. displayed a significant plateau in the progression of clinical signs before week 2 (11–14 dpi for 1.5 μg mice and 13–14 dpi for 3 μg mice). After day 14, the clinical score immediately caught up with vehicle treated mice (Figure 5B). No difference was detected in body weight loss between hMANF and vehicle treated mice (Figure 5C). As shown in earlier Figures 1D–F, EAE mice show decreased performance and mobility as compared to healthy controls at 7 dpi, just before the onset of clinical signs. EAE mice that were treated with either dose of hMANF showed a significantly better capability to remain on a rotarod (Figure 5D) at 7 dpi. Similarly, both doses of hMANF significantly increased distance traveled and vertical counts performed during open field-testing at 7 dpi (Figures 5E,F). No effect on rotarod or open field performance was detected at 14–28 dpi.

## Discussion

This study further showed that the intravenous administration of dex to MOG_3__3__–__5__5_/CFA induced mice postpones motor function loss in early stages of the disease. Moreover, dex was efficient at suppressing histological and immunological features of EAE, including the degeneration of myelin, axon loss and immune cell infiltration as well as the proinflammatory activation of microglia and astrocytes. Demyelination and the resulting axonal degeneration (Papadopoulos et al., 2006) of the CNS is invariably linked to immune cell infiltration in the EAE model of MS (Yednock et al., 1992; Ransohoff, 2012). Dex is believed to inhibit this process by reducing the number of circulating immune cells (Nguyen et al., 1997; Chen et al., 2006). This is further validated by our results, which showed that motor function loss, demyelination, proinflammatory glial activation and axonal degeneration in EAE mice treated with dexamethasone mirrored the progression of immune cell infiltration in the lumbar spinal cord (Figures 1–3).

Interestingly, our results showed that dex-treated EAE mice eventually reached similar clinical scores, axonal degeneration and spinal cord neuroinflammation, as vehicle-treated mice. This finding differs from several other studies, which generally show a more sustained neuroprotection from treatment with dex in EAE (Donia et al., 2010; Dos Santos et al., 2019). The difference may be due to different methods of administration, as dex is usually given i.p and not i.v., though it is not clear why intravenously given dex would lose effectiveness. The increased bioavailability in intravenous administration may lead to increased development of glucocorticoid resistance in circulating immune cells. Glucocorticoid resistance reduces the therapeutic effect of exogenous dex, likely via a downregulation of glucocorticoid receptors in target cells (Gold et al., 2012). The development of glucocorticoid resistance is an important factor in the clinical treatment of neuroinflammatory conditions with glucocorticoids such as dexamethasone, and EAE may be a practical model for studying its development (Hoepner et al., 2019). Alternatively, it is possible that intravenously administered dex is processed and excreted out of the body faster than in ip. reducing its therapeutic efficiency as EAE gains severity.

NTFs are abundantly expressed in a variety of cell types in the brain (Poo, 2001), and play an important role in both neurons and other cells of the CNS. The function and role of neurotrophic factors has been studied in both EAE mice and human MS patients. Reducing CNTF-production has led to poorer outcomes in EAE experiments (Linker et al., 2002), and the major neurotrophic factor BDNF has been reported to be highly expressed in infiltrated immune cells in MS patient brain tissue (Stadelmann et al., 2002). While glucocorticoids such as dexamethasone are generally though to play an anti-inflammatory role in EAE, dexamethasone has also been found to promote TrkB signaling, a known action mechanism of BDNF (Jeanneteau et al., 2008; Casarotto et al., 2021). Accordingly, treatment with dex has showed trophic effects on neurons both *in vivo* and *in vitro*, suggesting that in addition to its immunosuppressive abilities, dex can activate neurotrophic factor signaling in EAE.

In our study, we focused on the time-dependent expression of the novel neurotrophic factor MANF in EAE. We found that MANF was strongly expressed in the white matter of spinal cords in EAE mice, and that expression of MANF significantly decrease in a time-dependent manner in EAE. This decrease was in line with both the decrease in myelination, and the infiltration of immune cells in EAE mice (Figure 4). Interestingly, expression of MANF in spinal cord white matter were significantly increased by dex-treatment in early stages of EAE. This increased expression was highly co-localized with MBP-positive myelinated axons, the main targets of autoimmunity in EAE, while the colocalization of MANF with MBP was reduced along with MANF-levels during the peak of the disease. This poses the interesting question of whether this decrease in axonal MANF is a cause or result of axonal demyelination and degeneration.

As mentioned before, MANF is generally associated with activation of the UPR due to intense ER stress (Voutilainen et al., 2015). The UPR is an adaptive response to the stress caused by an accumulation of misfolded proteins in the ER of affected cells. It triggers both protective and apoptotic signals, and contributes to neurodegeneration in both MS and its disease models (Stone and Lin, 2015). Dex has an interesting relationship with ER stress and the UPR. In glomerular diseases such as nephrotic syndrome, it seems to reduce ER stress as a result of binding to glucocorticoid receptors (Fujii et al., 2006), whereas in a mouse model of asthma treatment, dex did not affect ER stress levels (Kim and Lee, 2015). In a murine glaucoma model topical eye treatment with dex can even be used to induce ER stress to an extent that leads to axonal degeneration (Zode et al., 2014). No results associating dex with the UPR in EAE models have currently been published. In this study we focused on the glucose-regulated protein 78 kDa (GRP78) to analyze the activation of the UPR in a time-dependent manner in EAE. GRP78, also known as Binding immunoglobin Protein (BiP), is an ER-resident protein that plays a critical role in the ER stress response (Casas, 2017). Although the mechanisms for the regulation and secretion of MANF are not completely understood, in Neuro2a cells MANF has been shown to be regulated in the ER by GRP78 (Oh-Hashi et al., 2012). In this report, we found that while expression of GRP78 was elevated in both EAE groups at the beginning of the experimental period, it was not affected by treatment with dex. Based on this observation, it seems that while an increase in MANF-expression is a result of treatment with dex during EAE, it is not linked to an increase in ER stress or activation of the UPR. In addition to its effects on ER stress, MANF is known to regulate immune cell activation (Neves et al., 2016), and has recently been discovered to be capable of suppressing NF-κB signaling by binding to neuroplastin on the cell membrane (Yagi et al., 2020). Thus, increasing MANF-expression may be another way for dex to regulate immune responses.

In order to test whether MANF itself can be therapeutic in EAE, we gave EAE-induced mice hMANF intravenously. This led to improved performances in behavioral tests at 7 dpi, before any signs of paralysis had manifested. Interestingly, the therapeutic effect of MANF on the clinical score was limited to 11–14 dpi, during which treatment with MANF halted the progression of paralysis. As a 20 kDa protein, MANF has poor bioavailability to the CNS. As the onset of EAE is associated with a significant increase in blood-brain- barrier (BBB) permeability (Wolburg et al., 2003; Bennett et al., 2010), it is possible that the breakdown of the endothelial BBB allows for a therapeutic time window during which administered exogenous MANF can access the CNS. This would need to be verified by further studies, as it doesn’t explain the positive behavioral effects before onset of paralysis, when immune cell infiltration is still scarce. The timing could also be explained by other factors, such as MANF having an anti-inflammatory effect on peripheral immune cells during the onset of the disease.

In summary, we proved the neuroprotective effect of the glucocorticoid dex in EAE mice immunized with MOG_3__5__–__5__5_ in a time-dependent manner. We showed that in the early stage of EAE, dex delays motor dysfunction and clinical disease onset. Additionally, treatment with dex postponed general histological features of EAE, including demyelination, axonal loss, immune cell infiltration, and neuroinflammatory glial cell activation. Interestingly, we observed an increased expression of MANF lumbar spinal cord myelinated axons during EAE, which was strongly elevated by dex in early stages of the disease. We then showed that intravenously injected hMANF reduced behavioral dysfunction and inhibited the progression of clinical signs at the early stage of the disease. These results indicate that MANF may play an important role in EAE, and should be considered a potential drug candidate in the treatment of MS. A better understanding of the interplay between MANF and neuroinflammatory responses is needed for the study of neuroinflammatory diseases such as MS.

## Data Availability Statement

The raw data supporting the conclusions of this article will be made available by the authors, without undue reservation.

## Ethics Statement

All experiments were undertaken with Animal license ESAVI/1229/04.10.07/2018, other permits were acquired from the National Animal Experimental Board ELLA, and mice were handled in accordance with all European Union guidelines and directives regarding the use of animals in scientific studies.

## Author Contributions

JN, TK, and MHV designed the study and wrote the manuscript. JN and TK conducted animal experiments, performed IHC, and analyzed the data. All authors contributed to the article and approved the submitted version.

## Conflict of Interest

The authors declare that the research was conducted in the absence of any commercial or financial relationships that could be construed as a potential conflict of interest.

## Supplementary Material

Supplementary Figure 1 Mean body weight for each experimental group during EAE. Mice were weighed each day during the study regarding the effect of dexamethasone in EAE, and mean group weights were used to calculate the dose of dexamethasone. Mean ± SEM, *n* = 7–26. Data from 4 repeated experiments.

Supplementary Figure 2 A single dose of dexamethasone did not affect MANF and MBP in naïve mice. **(A)** Diagram of the experiment design. All mice received dexamethasone (100 μg) or vehicle (PBS) intravenously (i.v. tail vein). Mice were transcardially perfused at an interval of 0.5, 1, 3, and 6 h. **(B)** Quantification of MANF and MBP area in each time point. Mean ± SEM. *n* = 5–6 per each group. **(C)** Representative fluorescence images of MBP (Green), MANF (Magenta), and DAPI (Blue) in the white matter of mouse spinal cords at 0.5, 1, 3, and 6 h. Scale bars: 10 μm. Data from a single independent experiment.

Supplementary Figure 3 GRP78 in the white matter of mouse spinal cords was not affected by dex-treatment in the EAE mouse model. Spinal cord sections were collected from EAE + Veh and dex-treated EAE mice at 7, 14, 21, and 28 dpi, in order to investigate whether GRP78 is regulated by dexamethasone or EAE progression in a time-dependent manner. **(A)** Representative fluorescence images show the expression of GRP78 (Red) and DAPI (Cyan) in the white matter of mice spinal cords. Enlarged images are taken from boxed areas from merged images. **(B)** Quantification of GRP78 area in each time point. Mean ± SEM. *n* = 5–8 per each group. Scale bars: 25 μm for GRP78/DAPI, 10 μm for enlarged images. Data from 4 repeated experiments.
