# Supplementary figures and images for: MANF Is Neuroprotective in Early Stages of EAE, and Elevated in Spinal White Matter by Treatment With Dexamethasone
Source: Front Cell Neurosci. 2021 Jul 7;15:640084. doi: 10.3389/fncel.2021.640084 (PMC8292680; doi:10.3389/fncel.2021.640084)

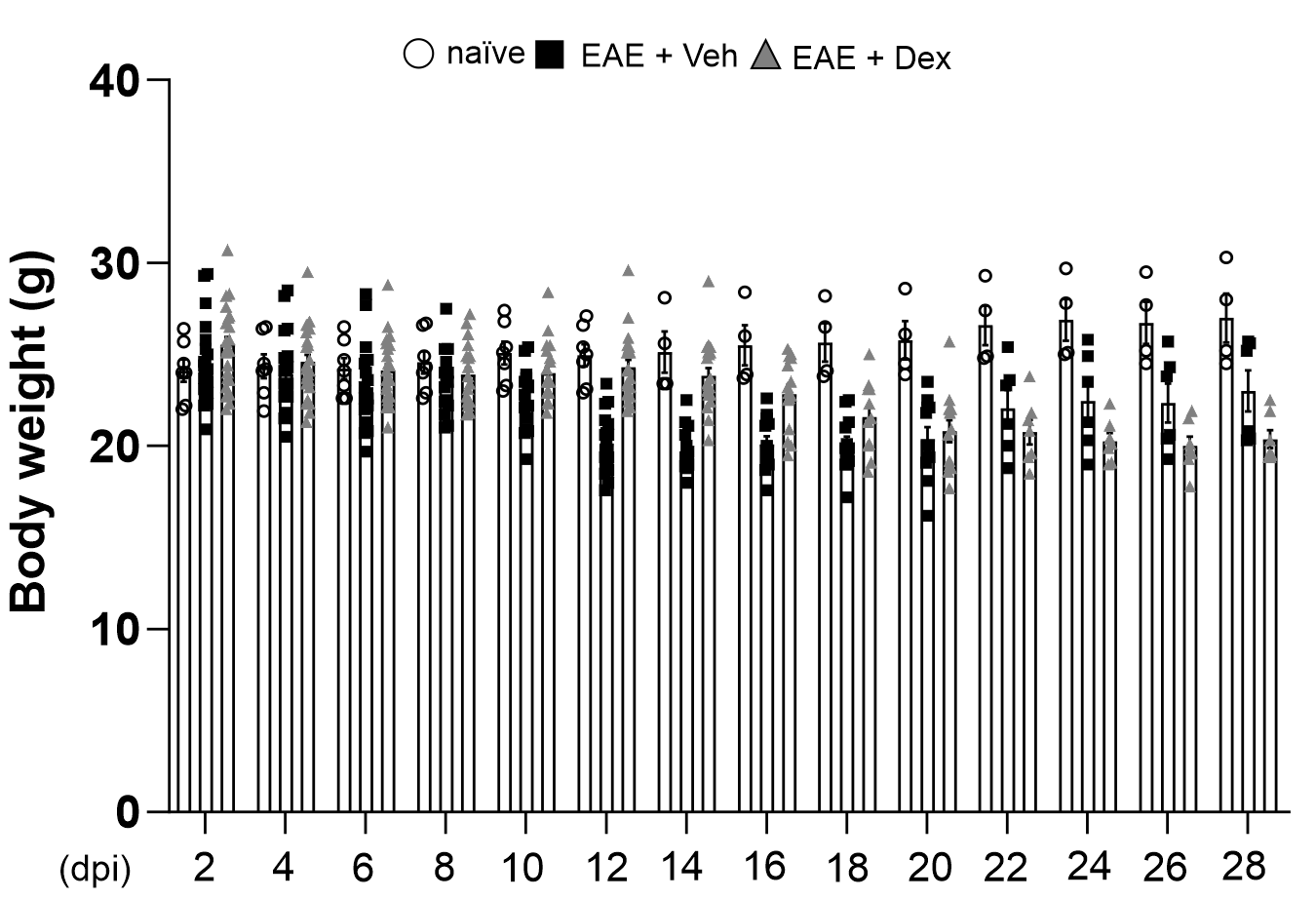

Supplement: Supplementary Figure 1 — Mean body weight for each experimental group during EAE. Mice were weighed each day during the study regarding the effect of dexamethasone in EAE, and mean group weights were used to calculate the dose of dexamethasone. Mean ± SEM, n = 7–26. Data from 4 repeated experiments. [file Image_1.tif]

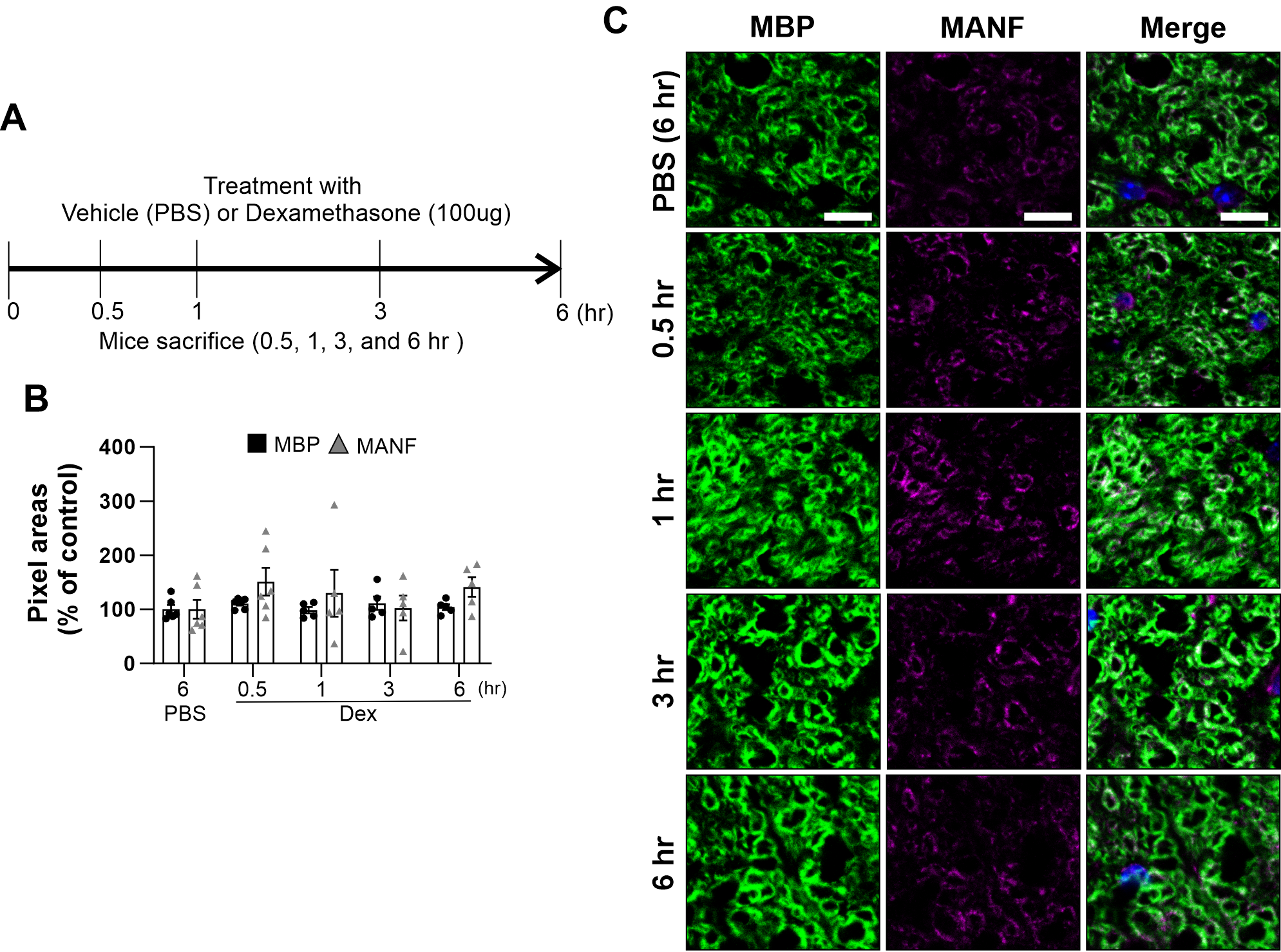

Supplement: Supplementary Figure 2 — A single dose of dexamethasone did not affect MANF and MBP in naïve mice. (A) Diagram of the experiment design. All mice received dexamethasone (100 μg) or vehicle (PBS) intravenously (i.v. tail vein). Mice were transcardially perfused at an interval of 0.5, 1, 3, and 6 h. (B) Quantification of MANF and MBP area in each time point. Mean ± SEM. n = 5–6 per each group. (C) Representative fluorescence images of MBP (Green), MANF (Magenta), and DAPI (Blue) in the white matter of mouse spinal cords at 0.5, 1, 3, and 6 h. Scale bars: 10 μm. Data from a single independent experiment. [file Image_2.tif]

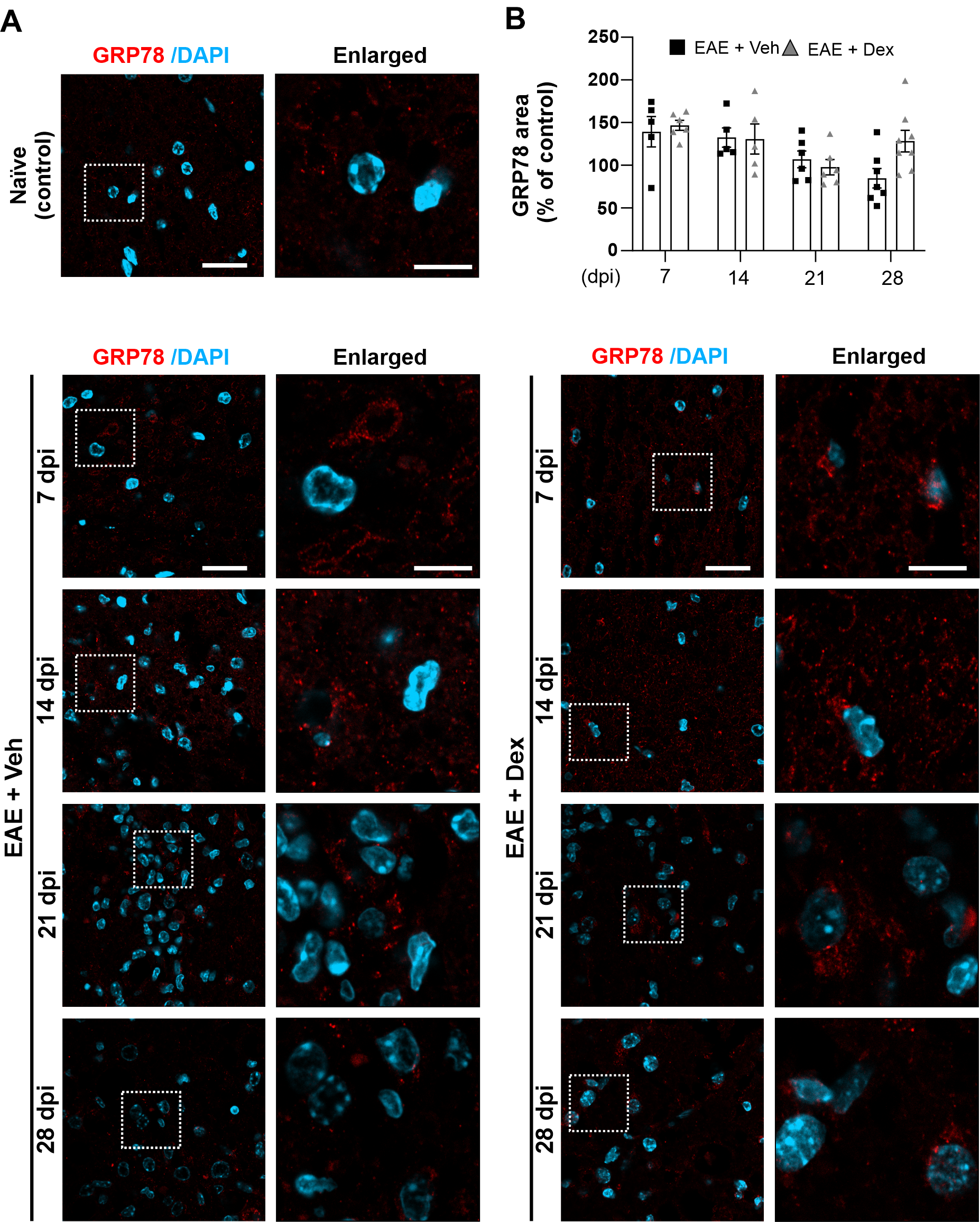

Supplement: Supplementary Figure 3 — GRP78 in the white matter of mouse spinal cords was not affected by dex-treatment in the EAE mouse model. Spinal cord sections were collected from EAE + Veh and dex-treated EAE mice at 7, 14, 21, and 28 dpi, in order to investigate whether GRP78 is regulated by dexamethasone or EAE progression in a time-dependent manner. (A) Representative fluorescence images show the expression of GRP78 (Red) and DAPI (Cyan) in the white matter of mice spinal cords. Enlarged images are taken from boxed areas from merged images. (B) Quantification of GRP78 area in each time point. Mean ± SEM. n = 5–8 per each group. Scale bars: 25 μm for GRP78/DAPI, 10 μm for enlarged images. Data from 4 repeated experiments. [file Image_3.tif]
